# The Mechanical Footprint of Dual Rinse 1‐Hydroxyethane 1,1‐Diphosphonate on Fast‐Set Mineral Trioxide Aggregate Microhardness and Dislocation Resistance

**DOI:** 10.1155/tswj/5210867

**Published:** 2026-04-08

**Authors:** Sahar Akbari Iraj, Anahita Kaboli, Noushin Shokouhinejad

**Affiliations:** ^1^ Department of Orthodontics, School of Dentistry, Shahid Beheshti University of Medical Sciences, Tehran, Iran, sbmu.ac.ir; ^2^ School of Chemistry, Khajeh Nasir Toosi University of Technology, Tehran, Iran; ^3^ Dental Research Center, Dentistry Research Institute, Tehran University of Medical Sciences, Tehran, Iran, tums.ac.ir; ^4^ Department of Endodontics, School of Dentistry, Tehran University of Medical Sciences, Tehran, Iran, tums.ac.ir

**Keywords:** calcium silicate–based cements, Dual Rinse HEDP, microhardness, push-out bond strength test, root canal irrigants

## Abstract

**Objectives:**

This study evaluated the effects of various root canal irrigation protocols on the surface microhardness and push‐out bond strength of a fast‐set hydraulic calcium silicate–based cement at two different stages of hydration.

**Materials and Methods:**

Standardized 3‐mm‐thick root slices with uniform lumens were filled with RetroMTA (BioMTA, Seoul, Korea). The slices were randomly assigned to two setting intervals (1 or 14 days), after which their upper surfaces were irrigated using one of several protocols, including 2% NaOCl, 5.25% NaOCl, Dual Rinse HEDP mixed with either 2% or 5.25% NaOCl, 2% or 5.25% NaOCl followed by EDTA, or normal saline (as a control). The dislocation resistance of RetroMTA was then measured using the push‐out bond strength test. For surface microhardness assessment, RetroMTA‐filled polymethyl methacrylate molds were exposed to the same irrigation protocols, and their surface microhardness was measured using the Vickers microhardness test. The data were analyzed using one‐way and three‐way ANOVA tests.

**Results:**

One‐way ANOVA showed that neither the 1‐day nor the 14‐day RetroMTA groups differed significantly from the control group in push‐out bond strength or surface microhardness (*p* > 0.05). Three‐way ANOVA demonstrated that no two‐way or three‐way interaction effects were statistically significant. Moreover, NaOCl concentration, chelator type, and the time interval between RetroMTA placement and irrigation had no significant effects on push‐out bond strength or surface microhardness (*p* > 0.05).

**Conclusion:**

Under the limitations of this experimental study, no significant differences were detected among the effects of Dual Rinse HEDP mixed with either 2% or 5.25% NaOCl, NaOCl alone, or NaOCl followed by 17% EDTA on the push‐out bond strength or surface microhardness of 1‐day and 14‐day RetroMTA samples.

## 1. Introduction

Sodium hypochlorite (NaOCl), due to its remarkable antimicrobial properties and ability to dissolve organic materials, is the most commonly used root canal irrigant [1]. However, it cannot remove the mineral component of the smear layer [2]. Therefore, a chelating agent such as ethylenediaminetetraacetic acid (EDTA) is usually used in addition to sodium hypochlorite. Intermittent use of EDTA presents disadvantages, including limited smear‐layer removal in the apical third [3] and a reduction in NaOCl’s available chlorine when the two solutions interact, which diminishes NaOCl’s antimicrobial and tissue‐dissolving efficacy [4].

1‐Hydroxyethane‐1,1‐diphosphonic acid (HEDP), commonly referred to as etidronic acid, is a biocompatible chelator that has recently been introduced under the brand name Dual Rinse HEDP (Medcem, Weinfelden, Switzerland) [5]. Due to the compatibility of HEDP with NaOCl, their mixture can be utilized as a single root canal irrigant, maintaining the properties of both during canal preparation [6, 7]. Continuous chelation with HEDP–NaOCl mixtures has been shown to be less damaging to dentin than the conventional sequence of NaOCl followed by EDTA [8].

Iatrogenic perforations of the root are accidents that may occur during root canal preparation and jeopardize the success of the treatment. Immediate repair of root perforations is essential to prevent contamination of the periodontal ligament [9]. Therefore, perforation repair material may be exposed to various root canal irrigants during subsequent root canal preparation.

Hydraulic calcium silicate–based cements (HCSCs), including mineral trioxide aggregate (MTA), are commonly used for various dental procedures such as root‐end filling, root perforation repair, pulp capping, apexogenesis, and apexification. The appropriate material for perforation repair should be biocompatible and create a strong seal at the site of the perforation [10]. Although ProRoot MTA (Dentsply, Tulsa, OK, USA) has revolutionized endodontics, it presents several drawbacks, including handling challenges, extended setting times, and a risk of tooth discoloration. Consequently, various types of HCSCs were developed to overcome these limitations [10]. The radiopacifier used in some HCSCs, bismuth oxide, has been linked to tooth discoloration [11]. RetroMTA (BioMTA, Seoul, Korea) comprises calcium carbonate, silicon oxide, aluminum oxide, and a calcium zirconia complex. It is a fast‐setting MTA with reduced discoloration potential, likely due to replacing bismuth oxide with zirconium oxide as the radiopacifier [12]. The zirconia complex in RetroMTA contributes to its shorter setting time [13, 14].

The hydration of HCSCs progress over time as the materials react with water and hardens. Sarkar et al. [15] showed that teeth filled with MTA and stored in synthetic tissue fluid for 2 months produced an adherent interfacial layer at the dentin wall that resembled hydroxyapatite in composition. Reports also indicate that the push‐out bond strength of MTA increases markedly within the first 3 days and continues to rise, though more moderately, by Day 21 [16].

When used as perforation repair materials, HCSCs can undergo physical and chemical alterations upon exposure to irrigants. For instance, 17% EDTA disrupts cement hydration and reduces its microhardness, biocompatibility, and flexural strength [17, 18]. Furthermore, it has been demonstrated that interaction with different root canal irrigants during the setting phase of HCSCs may impact the push‐out bond strength of perforation repair material [19, 20].

Since the Dual Rinse HEDP is a new irrigation solution, its impact on the physical properties of various types of HCSCs, such as surface microhardness and push‐out bond strength, is limited. Therefore, this study was designed to investigate the effects of various root canal irrigation protocols, including a mixture of HEDP with different concentrations of sodium hypochlorite (2% and 5.25%) compared with 2% NaOCl, 5.25% NaOCl, 2% NaOCl, or 5.25% NaOCl followed by 17% EDTA on the surface microhardness and push‐out bond strength of RetroMTA at two different stages of hydration.

## 2. Materials and Methods

### 2.1. Push‐Out Bond Strength

Extracted single‐rooted human teeth, all with straight roots, were selected and stored in 0.5% chloramine‐T at 4°C. The midroot dentin of each root was sectioned perpendicular to the long axis to produce slices of 3.00 ± 0.05‐mm thickness using a water‐cooled low‐speed diamond saw on a precision cutoff machine (Mecatome, Presi, France) to obtain a total number of 196 root slices. Gates Glidden burs (sizes 2–5) were used to standardize lumen diameter to 1.3 mm (Dentsply Maillefer, Ballaigues, Switzerland).

The prepared slices were placed on the glass slab. RetroMTA was prepared according to the manufacturer’s instructions and placed in the lumens, with the lower surface on blood‐soaked gauze and the upper surface covered with a cotton pellet moistened with normal saline to simulate clinical conditions. The whole fresh human blood used in this study was obtained from healthy volunteers who provided informed consent. This study was approved by the Ethics Committee of Tehran University of Medical Sciences (Ethics code: IR.TUMS.DENTISTRY.REC1401.152). After that, the root slices were randomly divided into 2 groups of 98 according to the different setting times: 1 day or 14 days. During the setting process, the specimens were stored in fully saturated humidity at 37°C.

Afterward, each of the 1 day and 14 day groups was randomly divided into 7 groups of 14 according to the irrigation protocols: 2% NaOCl, 5.25% NaOCl, Dual Rinse HEDP combined with 2% NaOCl, Dual Rinse HEDP combined with 5.25% NaOCl, 2% NaOCl followed by 17% EDTA, 5.25% NaOCl followed by 17% EDTA, or normal saline (as a control). In groups containing Dual Rinse HEDP, one capsule of the sustained‐release powder was mixed with 10 mL of either 5.25% NaOCl or 2% NaOCl, in accordance with the manufacturer’s instructions, and used immediately. The upper surface of RetroMTA was exposed to HEDP–NaOCl mixtures or NaOCl alone for 30 min, with the solutions refreshed every 5 min. EDTA was applied for 3 min without renewal. Then the upper surface of the RetroMTA was rinsed with 1 mL of normal saline.

To perform the push‐out bond strength test, the upper surface of cement was loaded with a 1 mm diameter cylindrical stainless‐steel punch. Loading was performed on a universal testing machine (Z050, Zwick/Roell, Ulm, Germany) at a speed of 1 mm/min. The maximum force applied to the cement before debonding was recorded in Newton (N).

Bond strength (MPa) was calculated by dividing the failure load by the adhesion area, determined from the formula 2*π* *r* *h*, where *π* is the constant 3.14, *r* is the radius of the root canal, and *h* is the thickness of the root slice in millimeters.

After the push‐out bond strength testing, the root slices were examined under × 25 magnification to determine the failure mode: adhesive (at the cement/dentin interface), cohesive (within the cement), or mixed failure (both adhesive and cohesive failure mode) (Figure 1).

FIGURE 1Various failure modes: (a) adhesive, (b) cohesive, and (c) mixed.

**Figure:**
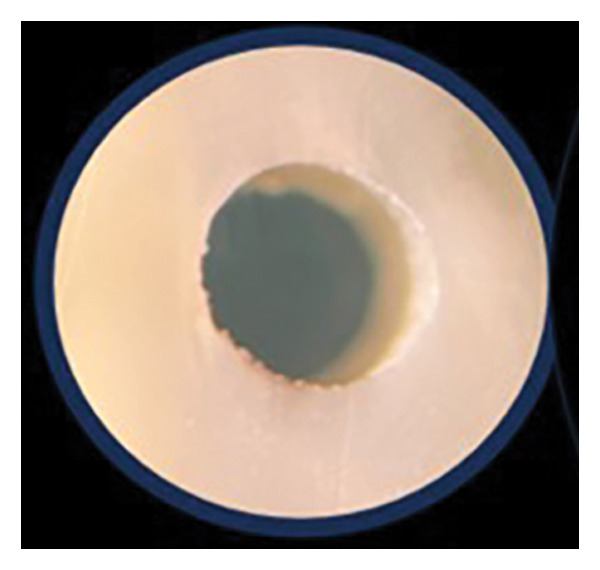
(a)

**Figure:**
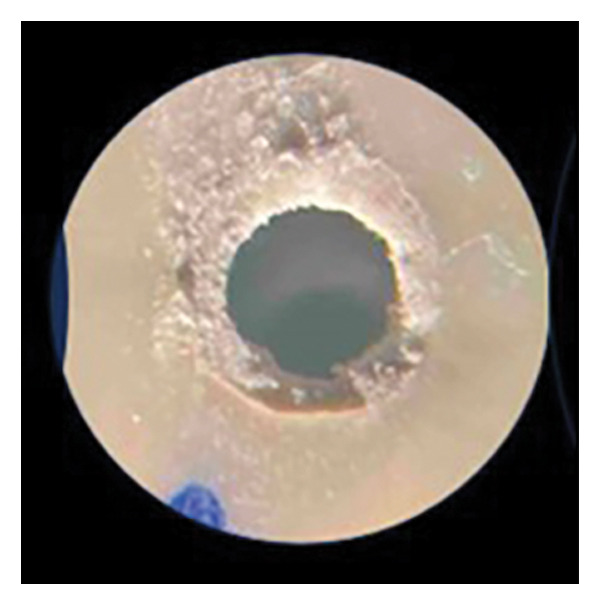
(b)

**Figure:**
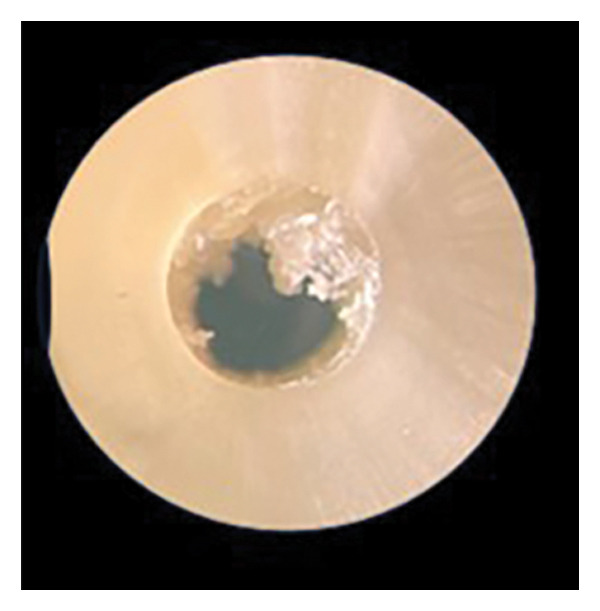
(c)

### 2.2. Surface Microhardness Measurement

To assess surface microhardness of RetroMTA exposed to different protocols of irrigation, the cement was mixed following the manufacturer’s instructions and placed in the cylindrical molds made of polymethyl methacrylate (*n* = 196), featuring an internal diameter of 6 mm and a height of 4 mm, in accordance with ASTM E384 standards. The lower surface of the cements was exposed to blood‐soaked gauze and a moist cotton pellet was placed on the upper surface. Subsequently, the specimens were randomly divided into two groups each containing 98 samples according to the different setting times: 1 day and 14 days. Throughout the setting process, the specimens were maintained in a fully saturated humid environment at 37°C.

The 1‐day and 14‐day experimental groups were each randomly divided into seven subgroups (*n* = 14), following the methodology specified for push‐out bond strength testing. Finally, the Vickers microhardness test was performed on the upper surfaces of samples that had been exposed to irrigation solutions. To assess surface microhardness, a square‐based pyramid indenter with a vertex angle of 136° applied a force of 300 g for 10 s onto the cement surface. Before microhardness testing, the irrigated surfaces were polished with consecutive use of wet silicon carbide sandpapers (#1000, #2000, and #3000 grit) to ensure smoothness and accurate indentation measurements. Three indentations were made on the polished surface of each sample at separate locations. The microhardness value of each specimen was calculated as the average of three indentations. The Vickers microhardness value was calculated based on the following formula: Vickers hardness number = 1.854 × (*F*/*d*2), where *F* is the load in kilogram‐force and *d* is the mean of the two diagonals produced by the indenter in millimeters.

Comparison of push‐out bond strength between the experimental groups and the control group was performed using one‐way ANOVA. After exclusion of the control group, a three‐way ANOVA was conducted to assess the main and interactive effects of NaOCl concentration, chelator type (HEDP, EDTA, or no chelator), and the time interval between RetroMTA placement and root canal irrigation (1 or 14 days).

## 3. Results

The mean push‐out bond strength and microhardness values are shown in Table 1.

**TABLE 1:** Mean (± standard deviation) push‐out bond strength and Vickers microhardness values.

Irrigation solution	Push‐out bond strength mean ± SD (MPa)		Microhardness mean ± SD (kg/mm^2^)	
	1 day (*n* = 14)	14 days (*n* = 14)	1 day (*n* = 14)	14 days (*n* = 14)
2% NaOCl	2.345 (0.637)	2.472 (0.921)	36.584 (3.796)	34.849 (6.744)
5.25% NaOCl	2.438 (0.916)	2.541 (0.683)	34.612 (3.236)	34.655 (3.209)
2% NaOCl + HEDP	2.485 (0.540)	2.182 (1.001)	34.827 (3.345)	36.356 (4.210)
5.25% NaOCl + HEDP	2.120 (0.684)	2.256 (0.597)	35.363 (5.963)	37.568 (4.230)
2% NaOCl/EDTA	2.316 (0.971)	2.240 (0.651)	36.959 (8.365)	36.096 (8.293)
5.25% NaOCl/EDTA	2.266 (0.939)	2.279 (0.871)	34.496 (4.698)	35.755 (4.005)
Normal saline	2.291 (0.704)	2.420 (0.552)	35.646 (6.015)	36.944 (6.641)

One‐way ANOVA revealed that neither the 1‐day nor the 14‐day RetroMTA groups differed significantly from the control group in terms of push‐out bond strength (*p* = 0.947 and *p* = 0.897, respectively). Similarly, no statistically significant differences in surface microhardness were observed between the 1‐day and 14‐day RetroMTA groups and the control group (*p* = 0.882 and *p* = 0.861, respectively).

Three‐way ANOVA demonstrated that none of the interaction effects, including both two‐way and three‐way interactions, were statistically significant. The concentration of NaOCl (2% vs. 5.25%) did not significantly affect the push‐out bond strength of RetroMTA samples (*p* = 0.862). Likewise, neither the type of chelating agent (HEDP, EDTA, or none) (*p* = 0.442) nor the time interval between RetroMTA placement and irrigation (1 day vs. 14 days) (*p* = 0.999) had a significant effect.

Regarding surface microhardness, the NaOCl concentration (*p* = 0.546), chelator type (*p* = 0.714), and the time interval between RetroMTA placement and irrigation (*p* = 0.648) likewise showed no statistically significant effects.

**FIGURE 2:**
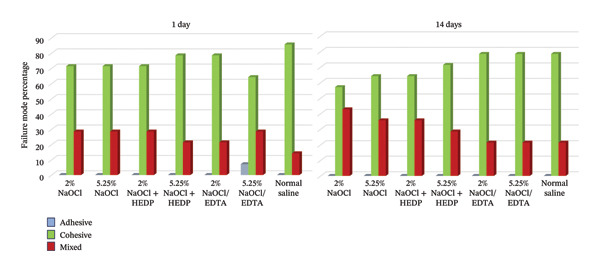
Frequency of three failure modes in different groups of 1‐day and 14‐day RetroMTA.

Inspection of the specimens after conducting the push‐out bond strength test showed that in all groups, the bond failure mode was mostly cohesive (Figure 2).

## 4. Discussion

In the present study, we investigated the effect of various irrigation solutions, including HEDP mixed with sodium hypochlorite at both full and diluted concentrations, on the surface microhardness and push‐out bond strength of RetroMTA. Sodium hypochlorite (NaOCl) was chosen as it is one of the most commonly used irrigants in endodontic treatment. The coronal surfaces of the RetroMTA specimens were exposed to the irrigation solutions, while the apical surfaces were placed in contact with blood‐soaked gauze to mimic clinical conditions of tooth perforation. In a number of studies, the effect of HEDP on the bond strength of RetroMTA was investigated in the time intervals of 1, 2, or 21 days after placing the cement [19, 21]. In this study, a 1‐day interval was selected as the minimum duration before the next patient visit following RetroMTA placement and continuation of endodontic treatment, with a 14‐day interval designated as the longer period.

To simulate clinical treatments, the RetroMTA coronal surfaces were treated with root canal irrigants for a total of 30 min, with the solutions being replaced every 5 min. EDTA was used for a shorter duration to be consistent with its typical clinical application protocol, which is applied at the end of root canal treatment to remove the smear layer.

In this study, Vickers microhardness and push‐out bond strength tests were conducted. The Vickers microhardness test has been frequently utilized in studies investigating the properties of HCSCs [22, 23]. Assessing the surface microhardness of HCSCs provides a partial understanding of the materials’ quality and their level of hydration progress, both critical factors for their setting [24, 25]. Furthermore, the resistance of a perforation repair material to dislodgement during the later stages of root canal treatment completion is an important characteristic of that material. The push‐out bond strength test is also commonly employed to evaluate the dislocation resistance of dental materials and to assess the bond strength of materials to dentin [19, 26–28].

According to the findings of the present study, NaOCl did not have significant impact on the bond strength of RetroMTA, and its effect was similar to that of normal saline. This finding is somewhat consistent with results of studies that did not reveal a significant difference in the push‐out bond strength of calcium silicate cements exposed to NaOCl compared with those exposed to distilled water or normal saline [19, 26]. On the other hand, some studies have shown conflicting results, indicating that NaOCl can either reduce or even increase the push‐out bond strength of various calcium silicate cements [29]. These discrepancies may stem from variations in exposure time, irrigant concentration, application method, or differences in the composition of the tested HCSCs. In this study, the samples were exposed to the irrigants at the coronal surface while simultaneously being exposed to blood from the apical side. In contrast, some studies have immersed cement samples in solutions [21, 30], which is different from clinical conditions. Furthermore, the type of HCSCs studied and differences in their constituent components may also result in differences in the physical and chemical properties of these materials, affecting their behavior when exposed to root canal irrigants.

The results of this study indicated that the push‐out bond strength of RetroMTA after 1 day and 14 days of setting, when exposed to a mixture of HEDP with either 2% NaOCl or 5.25% NaOCl, was similar to the bond strength of RetroMTA in contact with other irrigants. Our findings are consistent with those of Ulusoy et al. [21] who showed that the push‐out bond strength of RetroMTA after 2 days of setting and subsequent exposure to a mixture of HEDP and 2.5% NaOCl was comparable to that of 2.5% NaOCl followed by EDTA and 2.5% NaOCl alone. The findings of this study are somewhat in agreement with those of Rebolloso de Barrio et al. [26], who reported no significant differences in bond strength between ProRoot MTA and TotalFill when exposed to 5.25% NaOCl alone, a mixture of HEDP and 5.25% NaOCl, or normal saline. On the other hand, significant differences have been observed in the bond strength of some HCSCs after exposure to a mixture of HEDP and NaOCl, compared with NaOCl or EDTA [19, 26, 27]. The discrepancies in results may be due to differences in the types of HCSCs studied, the duration of exposure to the irrigants, or variations in methodology.

The findings of this study showed that none of the 1‐day RetroMTA groups exposed to the tested irrigants had a significantly different push‐out bond strength compared with the 14‐day groups. This result is to some extent consistent with the finding of a study [19] demonstrated that the bond strength of 1‐day Biodentine exposed to a mixture of NaOCl and HEDP did not differ significantly from that of 21‐day Biodentine. However, in the mentioned study, unlike the results related to Biodentine and also contrary to the findings of this study regarding RetroMTA, the bond strength of 21‐day ProRoot MTA was significantly higher than that of 1‐day ProRoot MTA. Differences in composition and setting kinetics may explain why some HCSCs reach substantial bond strength within the first 24 h. Supporting this, no significant difference was found between the bond strengths of RetroMTA at 1 and 14 days in this study and Biodentine at 1 and 21 days in Rebolloso de Barrio et al.‘s study [19]. The very short initial setting times of RetroMTA and Biodentine may account for the similar bond strength and hardness observed for the 1‐day and 14‐day cement groups exposed to various irrigants in this study, as well as for the 1‐day and 21‐day cement groups reported by Rebolloso de Barrio et al. [19].

The current study found that applying 17% EDTA for 3 min after a 30‐min rinse of the RetroMTA surface with 2% or 5.25% NaOCl does not result in a significant difference in bond strength compared with other root canal irrigants or normal saline. Some previous studies examining the effect of EDTA on the bond strength of calcium silicate cements used EDTA alone, without prior rinsing with NaOCl [19, 26, 31]. Because EDTA alone is seldom used clinically, these studies offer limited comparability to real‐world treatment protocols.

In the present study, the failure mode was predominantly cohesive. These findings are consistent with the studies which demonstrated that the failure pattern of HCSCs exposed to the HEDP mixed with NaOCl was mostly cohesive [26, 27]. However, Ulusoy et al. [21] showed that the failure mode of RetroMTA exposed to the mixture of Dual Rinse HEDP and NaOCl was mostly mixed. The low frequency of adhesive failure mode across all experimental groups indicates a robust interfacial bond between RetroMTA and the root dentin walls, across multiple irrigation protocols. The findings of this study are in agreement with studies showing that adhesive bond failure occurred in a very small number of HCSCs specimens following various irrigation procedures [27, 32]. Furthermore, the application of different irrigation procedures did not influence the push‐out bond failure mode of RetroMTA, consistent with the findings of Ulusoy et al. [21].

The current study indicated that the surface microhardness of RetroMTA after 1 day and 14 days of setting, when exposed to a mixture of HEDP with either 2% NaOCl or 5.25% NaOCl, was similar to that of RetroMTA in contact with other irrigants. The findings of this study are in contradiction with the results of the study by Butt and Talwar [23], who showed that 5.25% NaOCl reduces the surface microhardness of MTA after 1 day of setting. Chu et al. [22] also showed varying effects of NaOCl and EDTA on the surface microhardness of 1‐day and 8‐day samples depending on the type of calcium silicate cement examined. The findings of this study regarding the effect of HEDP on the surface microhardness of RetroMTA cannot be interpreted due to the lack of information on the impact of HEDP on the surface microhardness of HCSCs. Furthermore, direct comparison cannot be made due to differences in the study designs and the HCSCs examined. In clinical settings, several factors contribute to the ultimate outcome of endodontic treatments, necessitating further clinical trials to validate the findings of this study.

## 5. Conclusion

Under this experimental configuration, no significant differences were detected among the effects of Dual Rinse HEDP mixed with either 2% or 5.25% NaOCl, NaOCl alone, or NaOCl followed by 17% EDTA on the push‐out bond strength or surface microhardness of 1‐day and 14‐day RetroMTA samples. Given the differences in the conditions of in vitro and ex vivo studies compared with clinical conditions, the results of this study should be validated against well‐designed clinical studies.

## Funding

This study was funded and supported by the Dental Research Center, Dentistry Research Institute, Tehran University of Medical Sciences, Tehran, Iran (Grant no. 1402.1.133.65689).

## Conflicts of Interest

The authors declare no conflicts of interest.

## Data Availability

The data that support the findings of this study are available on request from the corresponding author. The data are not publicly available due to privacy or ethical restrictions.
